# Healthcare Service Utilisation of People Living With Non-Alzheimer’s Dementia: A Systematic Review

**DOI:** 10.1177/08919887251371725

**Published:** 2025-08-28

**Authors:** Anna Tjin, Leng Leng Thang, Harsharon Kaur Sondh, Robert Stewart

**Affiliations:** 1 Psychological Medicine, Institute of Psychiatry, Psychology & Neuroscience, 4616 King’s College London, London, UK; 2 Department of Japanese Studies, 37580 National University of Singapore, Singapore; 3 South London and Maudsley NHS Foundation Trust, Michael Rutter Centre, London, UK

**Keywords:** non-Alzheimer’s dementia, healthcare service utilisation, systematic review, dementia with lewy bodies, frontotemporal dementia, vascular dementia, Parkinson’s disease dementia, care needs

## Abstract

**Introduction:**

The global rise in dementia presents significant challenges for healthcare systems. While Alzheimer’s disease (AD) dominates dementia care, people with non-Alzheimer’s dementias (non-AD), such as dementia with Lewy bodies (DLB), frontotemporal dementia (FTD), vascular dementia (VD), and Parkinson’s disease dementia (PDD), often have distinct and unmet healthcare needs.

**Aim:**

This systematic review aimed to summarise evidence on healthcare utilisation (HCU) patterns and factors affecting care among people living with non-AD dementias.

**Methods:**

Following a PROSPERO-registered protocol (CRD42024568391), comprehensive searches of Embase, Ovid MEDLINE, Global Health, PsycINFO, and PubMed were conducted in February and June 2024. Peer-reviewed English-language studies reporting on HCU or its determinants in DLB, FTD, VD, or PDD were included. Reviews, case reports, grey literature, and studies without subtype-specific data were excluded. Quality was assessed using the Newcastle-Ottawa Scale.

**Results:**

Thirty-one studies (16 cohort; 10 cross-sectional, 4 case-description, and 1 chart review) were included. HCU varied by dementia subtype and was influenced by sociodemographic, cognitive, and clinical factors. Compared with AD, non-AD dementias had higher healthcare use and costs. PDD showed the highest inpatient, outpatient, and skilled nursing care use, driven by severe cognitive decline. DLB was linked to unplanned hospital admissions and frequent ambulance use, often due to falls and pneumonia. FTD resulted in extended hospital stays related to behavioural symptoms, while VD incurred high costs due to chronic comorbidities and long-term care needs.

**Conclusion:**

People with non-AD dementias have greater and distinct healthcare needs. Future research should develop standardised measures and tailored interventions to address their complex socioeconomic and clinical requirements.

## Plain Language Summary

A summary of global evidence on healthcare use among people with less common dementias and the factors influencing their use of healthcare services. Dementia affects millions of people worldwide, and most services and research focus on Alzheimer’s disease. However, there are other types of dementia (such as dementia with Lewy bodies, frontotemporal dementia, vascular dementia, and Parkinson’s disease dementia) that are less well known but can be just as serious. People with these less common types of dementia often have different symptoms and healthcare needs compared to those with Alzheimer’s disease. We wanted to better understand how people with these non-Alzheimer’s dementias use healthcare services. To do this, we looked at studies from around the world that reported how often people with these conditions visit hospitals, clinics, and other care settings. We also looked at what factors, such as age, memory problems, or other health issues, might affect how they use healthcare. Our review included 31 studies from different countries. We found that people with non-Alzheimer’s dementias often used more healthcare services than those with Alzheimer’s. For example, people with Parkinson’s disease dementia were more likely to be admitted to hospital or need nursing care. People with dementia with Lewy bodies often ended up in hospital unexpectedly due to falls or infections. Those with frontotemporal dementia had longer hospital stays because of behaviour changes. Vascular dementia was linked to long-term care needs due to other ongoing health problems like stroke or heart disease. Overall, we found that there is no one-size-fits-all approach. Each type of dementia has its own challenges and care needs. This means healthcare systems need to do more to adapt and provide the right care at the right time for people living with different types of dementia. We hope this research helps policymakers, caregivers, and health professionals improve care for people with all forms of dementia.

## Introduction

The global prevalence of dementia is growing rapidly, with an estimated 159 million people by 2050.^1^ Dementia has become one of the significant challenges to individuals, communities, healthcare systems, and societies.^2,3^ Amongst other priorities, there is an urgent need to address the complexity of needs associated with dementia diagnosis and management through a comprehensive and holistic approach. Unfortunately, multiple studies have identified a lack of coordination and fragmented healthcare system components supporting this population,^4,5^ highlighting the discrepancies between currently available services and effective high-quality dementia diagnosis and management provision.^4^ Furthermore, healthcare systems are often designed to cater for the majority. Alzheimer’s disease (AD), the most common (60%–70%) neurodegenerative disorder causing dementia,^6^ often shapes dementia services, potentially disadvantaging those with less common non-Alzheimer (non-AD) subtypes in terms of equitable access to and availability of support and services.^7^

One of the first steps in supporting the development of an inclusive dementia healthcare system is to understand the pattern of healthcare utilisation (HCU) for people with non-AD. HCU, in the context of this review, refers to the frequency, types, and quality of formal healthcare services. We focus exclusively on HCU rather than social or unpaid care, acknowledging that while the latter can exceed the costs of formal medical care, HCU data are more consistently available across studies and provide a foundational understanding of how health systems engage with this underrepresented population.

## Methods

This study is registered in PROSPERO (CRD42024501188). The PRISMA (Preferred Reporting Items for Systematic Reviews and Meta-analyses) guidance were applied to establish clear criteria and presentation of the review processes.^8^ The development of the methodology was informed by the systematic review guidance provided by Moola et al. (2015)^9^ and Munn et al. (2018).^10^

### Review Question and Objectives

This review addressed the question: *“What are the patterns, quality, and determinants of healthcare utilisation (HCU) among people with non-Alzheimer’s dementias (non-AD)?”* It systematically synthesised existing evidence to identify and describe the types and patterns of formal healthcare services accessed by this population, evaluate the quality of these services and their associated clinical outcomes, and examine socioeconomic and clinical factors influencing variations in HCU across dementia subtypes and settings. In doing so, it also sought to highlight gaps in the literature to inform future research and the development of more inclusive health systems. The review focused on the more common non-Alzheimer’s dementia subtypes, dementia with Lewy bodies (DLB), frontotemporal dementia (FTD), vascular dementia (VD), and Parkinson’s disease dementia (PDD), with the aim of improving understanding of these less-researched populations, identifying priorities for further investigation, and supporting efforts to strengthen health system inclusivity.

### Search Strategy

We searched Embase, Ovid MEDLINE, Global Health, PsycINFO, and PubMed for relevant studies without publication date restrictions. Although the primary focus of this review was on HCU patterns, we included cost-related search terms to recognise the role that economic factors can play in shaping access to and uptake of services. Barriers to care, such as affordability, cost of treatment, or variation in service provision across funding models, may influence patterns of healthcare use. However, given the broader scope of the review, economic evaluation-specific databases (e.g., NHS EED, EconLit) were not searched. This may have limited the identification of detailed cost-effectiveness data, which was not the primary objective of this review. To assess equity, we systematically identified and extracted data from studies that reported subgroup comparisons relevant to the dementia subtype (e.g., non-AD vs AD) or between individuals with dementia and comparable non-dementia populations. We recorded whether studies examined differences in service access, utilisation frequency, or outcomes and whether these factors were adjusted for indicators of clinical need such as age, disease severity, or comorbidities. Additionally, we focused on socioeconomic determinants of HCU, exploring how factors like income, education, and ethnicity influenced service access and utilisation. This approach enabled the synthesis of evidence related to horizontal equity (equal use for equal need) and vertical equity (appropriate variation in use based on differing needs).

The search strategy for EMBASE (Table 1) was revised for each database searched. The searches were conducted in February 2024 and were rerun in June 2024 to identify further eligible studies published in the review period. Inclusion criteria comprised the following: original research studies on HCU of people with non-AD over 18 years old (DLB, FTD, VD, PDD). Studies were included if they reported on patterns of HCU or made comparisons relevant to equity in access, provision, or outcomes. Studies investigating non-dementia participants, medical case studies, care directives, and economic cost without any separate description of HCU, non-English language, non-peer-reviewed studies, and any editorial, opinion, conference, and poster abstract without any original research findings were excluded.

**Table 1.** Search Criteria and Search Terms.

Population	Health service use
Exp dementia/ or exp dementia/ or exp alzheimers/ or exp lewy body/ exp frontotemporal dementia/	Exp health care/ or exp primary health care/ or exp health service/ exp hospital admission/ exp hospital utilization/ exp health care utilisation/ exp secondary health care
“Non-alzheimer* dementia*” OR “lewy bod*” OR “DLB” OR “LBD” OR “frontotemporal dementia” OR “FTD” OR “vascular dementia*” OR “dementia* vascular” OR “synucleinopathies”	“Health care*” OR “hospitali?ation” OR “memory clinic” OR “primary care” OR “secondary care” OR “speciali?ed care” OR “mental health service*” OR “utili?ation” OR “usage” OR “access” OR “deliver*” OR “barrier*” OR “facilitator*” OR “determinant*” OR “unmet need*” OR “care receipt*” OR “quality of life” OR “quality of service*” OR “cost*” OR “social determinants of health” OR “epidemiology” OR “driver*” OR “factor*” OR “characteristic*” OR “influencer*” OR “determinant*” OR “cause*”

### Study Selection

The inclusion and exclusion criteria were developed collaboratively by the research team. One researcher conducted the initial search and performed the title and abstract screening based on these agreed criteria. To ensure consistency and reduce potential bias, a second researcher independently reviewed a random sample of the screened studies and flagged any discrepancies for further discussion. Full-text screening was carried out by the same primary researcher. Following this, two researchers jointly conducted data extraction: one took the lead in extracting the data, while the other cross-checked it for accuracy and completeness. The entire research team subsequently reviewed the extracted data. Finally, a quality assessment was independently conducted by two researchers to strengthen the methodological rigour of the review.

Given that many studies drew on administrative databases, it is acknowledged that diagnoses of dementia were often assumed accurate without clinical verification, which may introduce potential misclassification and influence the reported patterns of healthcare utilisation.

### Quality Assessment

The Newcastle-Ottawa Scale (NOS) quality assessment scale for non-randomised studies, including case-control and cohort studies,^11^ was used to assess quality. Each study was rated utilising NOS on nine variables according to three categories (e.g., selection of groups, comparability of the groups, and outcome of interest). More points indicate less risk of bias and higher quality, ranging from good (≥7 points), fair (2-6 points), to poor (≤1 point).^11^ Although cost-related factors such as affordability and economic barriers were considered as drivers of HCU, a detailed examination of cost estimation methods was not the primary focus of this review. Therefore, a comprehensive economic quality assessment tool was not applied. The quality assessment was carried out with the outcome of interest being HCU. As we sought to be inclusive with evidence, the study quality is reported in the findings and factored into result interpretation.

## Result

**Figure 1.**
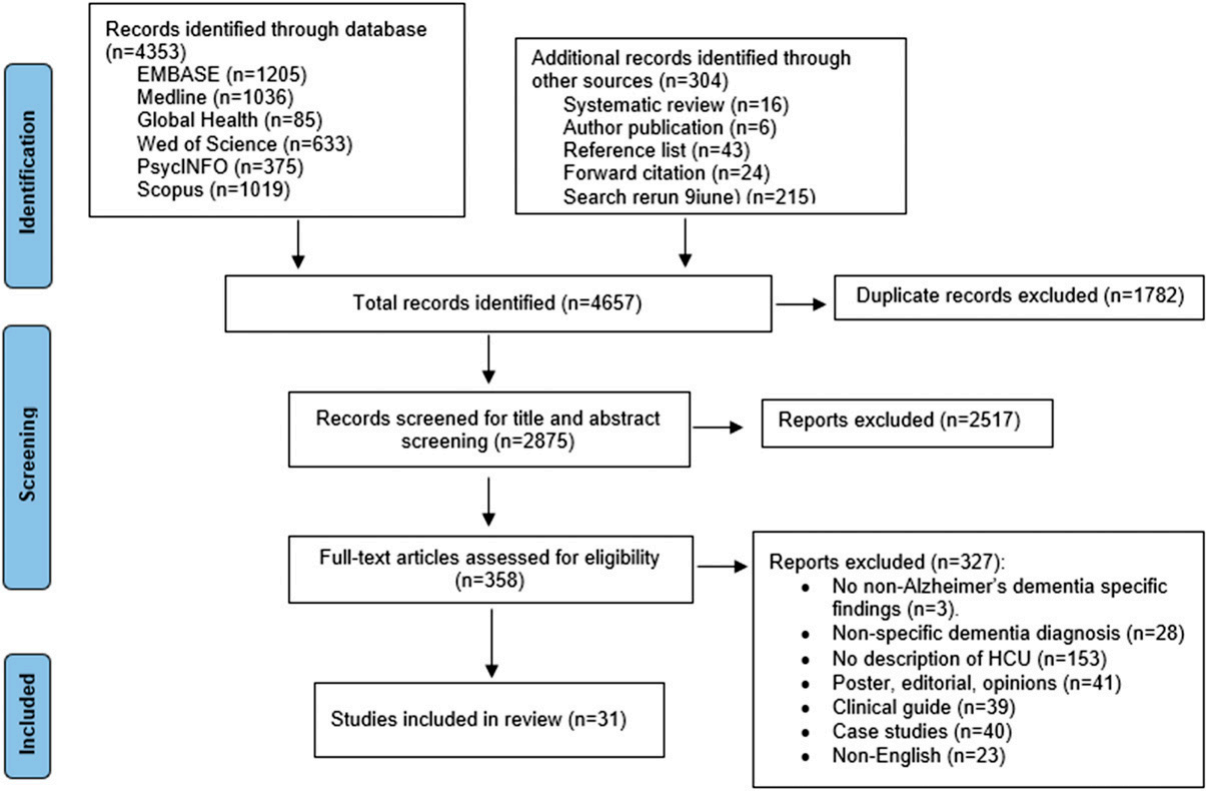
PRISMA flowchart.

The systematic literature search (Figure 1) resulted in 4353 articles. Additional records (n = 304) were identified through systematic reviews (n = 16), publications from included authors (n = 6), reference lists (n = 43), forward citations (n = 24), and search rerun (n = 215). After removing 1782 duplicates, 2875 articles went through title and abstract screening. A total of 358 articles were included in the full-text review.

Thirty-one studies met the inclusion criteria and were included in this systematic review (Table 2). Fifteen studies were rated good quality, 13 were fair, and three were poor. Of the included studies, six^12-16^ explicitly aimed to examine access to services or barriers to access, such as affordability, availability, or system-level obstacles. The remaining studies primarily focused on quantifying levels of service use (e.g., frequency of hospital admissions) without exploring underlying barriers. This distinction highlights the variability in how HCU was conceptualised across the literature.

**Table 2.** Studies Included in the Review.

First author (Year)	Population of interest (n)	Study design (setting)	Quality
Albrecht (2018)^12^	VD (1,520);DLB (204);FTD (153)	Cohort (PRD + POD)	Good
Bostrom (2007)^17^	DLB (34)	Cross-sectional (POD)	Fair
Chang (2015)^13^	VD (96);PDD (59)	Cohort (POD)	Fair
Chen (2019)^18^	DLB (10,869);VD (9,911);FTD (729)	Cross-sectional (POD)	Fair
Dauphinot (2021)^14^	VD (176);DLB (40);FTD (12);PDD (33)	Cost-description (POD)	Fair
Desai (2022)^15^	DLB (9,019);DLB (4,796);PDD (4,223)	Cross-sectional (POD)	Fair
Espinosa (2020)^19^	DLB (536)	Cohort (PRD + POD)	Good
Fereshtehnejad (2018)^20^	VD (10,923);DLB (1,177);PDD (842);FTD (857)	Cohort (PRD + POD)	Good
Fillit (2002)^21^	VD (240)	Cost-description (POD)	Fair
Forns (2022)^22^	PDD (49,004);DLB (15,263);FTD (4,382);VD (60,132)	Cohort (POD)	Good
Frazer (2023)^23^	PDD (136);VD (64);DLB (10)	Cross-sectional (PRD + POD)	Fair
Hanyu (2009)^24^	DLB (56)	Cohort (POD)	Good
Henderson (2019)^25^	VD (171);FTD (54);PDD (44);DLB (53)	Cost-description (POD)	Fair
Henderson (2022)^26^	FTD (54);PDD (44)	Cohort (POD)	Fair
Hill (2005)^27^	VD (678)	Cohort (POD)	Fair
Jaeger (2021)^16^	VD (47)	Cross-sectional (D)	Fair
Landi (1999)^28^	VD (78,434)	Cohort (POD)	Good
Llanes-Alvarez (2020)^29^	VD (607)	Cross-sectional (POD)	Fair
Matsuoka (2019)^30^	DLB (18);VD (23)	Cohort (POD)	Good
Mueller (2018)^31^	DLB (194)	Cohort (pre and POD)	Good
Murman (2002)^32^	PDD (85)	Cross-sectional (POD)	Fair
Murman (2003)^33^	DLB (15)	Cross sectional (POD)	Poor
Natalwala (2008)^34^	VD (283)	Cohort (POD)	Fair
Oesterhus (2020)^35^	DLB (91)	Cohort (POD)	Fair
Pisu (2021)^36^	VD (7,211)	Cohort (PRD + POD)	Fair
Ramos-Estebanez (2012)^37^	VD (122)	Cost-description (POD)	Fair
Sicras (2005)^38^	VD (63)	Cross-sectional (POD)	Fair
Spears (2019)^39^	DLB (117)	Cohort (POD)	Fair
Thoits (2020)^40^	VD (111);AD + VD (58)	Chart review (D + POD)	Fair
Zhu (2008)^41^	DLB (25)	Cross sectional (POD)	Fair
Zilkens (2009)^42^	VD (2,595);PDD (239);FTD (39)	Cohort (POD)	Good

*VD = Vascular Dementia; DLB = Dementia Lewy Body; PDD = Parkinson’s Disease Dementia; FTD = Frontotemporal Dementia.

*PRD = Pre-diagnosis; POD = Post-diagnosis; D = Diagnosis; PL = Palliative.

### Study Characteristics

The 31 studies (Table 3) originated from the USA (n = 15), United Kingdom (n = 4), Spain (n = 3), Sweden (n = 2), Japan (n = 2), Australia (n = 1), France (n = 1), Norway (n = 1), Taiwan (n = 1), and Brazil (n = 1). The articles were published between 1999 and 2023, with 15 published between 2019 and 2023. Sixteen of the studies were cohort studies, followed by cross-sectional (n = 10), cost-benefit analysis (n = 4), and chart review (n = 1). Twenty-three studies analysed HCU in the post-diagnostic phase, six studies analysed pre- and post-diagnostic, one included diagnostic and post-diagnostic, and one explored the diagnostic stage. Sample sizes ranged from 68 to 484,520 participants.

**Table 3.** Characteristics of Articles Included.

Variables	n
Country (continent)	
North America	14
Europe	12
South America	1
Asia	3
Australasia	1
Year of publication	
1999-2003	4
2004-2008	5
2009-2013	3
2014-2018	4
2019-2023	15
Study types	
Chart review	1
Cost-description study	4
Cross sectional study	10
Cohort study	16
Healthcare setting	
Diagnosis	1
Diagnosis and post diagnosis	1
Pre and post diagnosis	6
Post-diagnosis	23

There was a clear imbalance in the representation of dementia subtypes across the included studies, with Alzheimer’s dementia (AD) consistently overrepresented and non-AD comprising relatively small sample sizes. For instance, in Albrecht et al. (2018) (12), AD accounted for 10% (n = 3748) of the cohort, while VD (n = 1520), LBD (204), and FTD (n = 153) represented only 0.4%-4%. Similarly, Chen et al. (2019) (15) reported 23.2% with AD, compared to 4.4% LBD, 4.0% VD, and 0.3% FTD. Matsuoka et al. (2019) (30) showed a comparable distribution: 48.0% AD, 14.0% dementia with Lewy bodies (DLB), 18.0% VD, and 2.0% FTD.

## Determinants of Healthcare Utilisation

Findings across the included studies presented mixed evidence regarding the extent to which HCU patterns among non-AD are attributable to the diagnostic subtype. While some studies^13,40^ reported no statistically significant differences in HCU across dementia subtypes, others^15,22^ identified elevated utilisation and associated costs among VD, DLB, FTD, and PDD, which were frequently characterised by higher levels of comorbidity, frailty, falls, hallucinations, and neuropsychiatric symptoms. A range of biopsychosocial and contextual variables appeared to exert a substantial influence on healthcare use. Socioeconomic disadvantage, rurality, and ethnic minority status were associated with increased HCU and longer hospital stays,^36,42^ while comorbidities—including cardiovascular, cerebrovascular, metabolic, and psychiatric conditions,^12,25-27,30,33,36^ such as diabetes,^12-14,38^ hypertension and hypertensive heart disease,^22,38^ stroke,^38^ anxiety,^14^ and mood disorders^19^—were consistently linked with higher HCU. Clinical features such as fluctuating cognitive performance,^14,19^ lower functional autonomy level,^14,19^ delirium,^19^ motor symptoms,^19^ a higher number of medications,^14^ and caregiver burden^14^ also emerged as relevant determinants. Notably, several studies^30,40^ emphasised the predictive value of dementia severity and behavioural and psychological symptom burden, indicating that these clinical factors may better account for variations in HCU than diagnostic subtypes alone.

## Healthcare Service Utilisation by Dementia Subtype

Healthcare service utilisation varies across dementia subtypes, reflecting differences in clinical profiles and care needs. Patterns of emergency visits, hospitalisations, outpatient care, and long-term care differ notably between FTD, PDD, DLB, and VD. A summary of healthcare use by subtype is provided in Table 4.

**Table 4.** Healthcare Service Utilisation by Dementia Subtype.

Dementia subtype	Emergency department (ED) use	Primary care & outpatient visits	Hospitalisation & inpatient care	Long-term care	Other notable service use
Frontotemporal dementia (FTD)	Low incidence ED visits.^22^	Low overall HCU even with increased service use over disease duration.^18,26^	Low hospitalization rate; cost differences driven by hospitalisation rate and length of stay.^18^	-	Higher rates of behavioural and psychological symptoms.^22,26^
Parkinson’s disease dementia (PDD)	High rates of ED visits related to falls/fractures, pneumonia and acute delirium.^13,22^	Decline in dental care utilisation^20^; increased outpatient service use overtime^12,15,32^	High risk of hospital admission and longer hospital stays^13,32,33^	Increased use overtime^15,32^	Higher burden of cerebrovascular risk factors, stroke, hallucinations, and acute delirium episodes^13,22^
Dementia with lewy bodies (DLB)	High rates of ED visits, with greater medication use and ambulance service use.^15,17,18,22-24,33,39^	Increased outpatient visits, more GP consultations, and higher use of informal services^17,18,23^	Higher, earlier hospitalisation (87%vs69% in AD); 40% of admissions due to neuropsychiatric symptoms.^18,23,31,33,35,39^	High rates of formal long-term care use^33^	Complex clinical profile with high comorbidity burden; increased mortality mainly due to falls & bronchopneumonia.
Vascular dementia (VD)	Higher ED visits than AD (5.8 vs 4.2)^18^; increased ambulance use (6.5 vs 3.8).^18^	Greater primary care follow-ups (14.7 vs 8.8 visits), specialist visits, lab tests, and supplemental exams than AD.^12,18,36,38^	Higher hospitalisation rates (27%vs13%), longer stays (4.7 vs 2.4 days) than AD; nearly double hospital admissions and length of stay.^13,18,21,22,27-29,34,37,38,42^ Frequent admissions due to stroke, falls, fractures, pneumonia.^13,22,34,37^	High rates of nursing facility admissions and skilled nursing care.^12,22,27^	Highest burden of cerebrovascular/ cardiovascular comorbidities; cognitive impairment less predictive of hospitalisation risk.^12,13,21,22,27,28,38^

### Frontotemporal Dementia (FTD)

Eight studies include FTD, but only three reported findings specific to FTD. FTD often had the smallest sample size compared to other subtypes, ranging from 14%^12^ to less than 4%.^14,18,20,22,25,26,42^

Chen et al. (2019)^18^ and Henderson et al. (2022)^26^ reported that of diseases causing dementia, FTD was one of the lowest in terms of costs and HCU, even with a significantly increased use of services over the duration of their condition.^18,26^ Multiple studies report higher rates of behavioural and psychological disturbance,^18^ including delusions,^22^ apathy,^26^ disinhibition^26^ and obsessions.^26^ However, Forns et al. (2022)^22^ reported the lowest incidence rates for almost all events of interest in FTD, especially aspiration pneumonia infections and Emergency Department (ED) visits, and the lowest proportion of Medicare coverage (26.4%), with the highest proportion observed in VD (40.4%).

**Table 5.** Summary of Patterns of Healthcare Utilisation Categorised by Cost (Converted to USD).

Author (year)	Country (currency)	HCU	Time	DLB	AD	VD	PDD	FTD
Chen (2019)^18^	USA (USD)	MC,CC	Annual (mean)	22,514	13,935	21,002		14,853
Dauphinot (2021)^14^	France (EUR)	MC,TP	1y POD	21,682	11,073		22,471	
Henderson (2022)^26^	UK (GBP)	MC,CC,SC	Weekly	548	158	166	650	336
Desai (2022)^15^	USA (USD)	MC,ME	Annual (mean)	15,903			21,040	
Henderson (2019)^25^	UK (GBP)	MC,SC,CC,ME	3m		4415		10,911	
Murman (2003)	USA (USD)	MC,CC,ME	Annual (mean)	25,311	11,557			
Zhu (2008)^41^	USA (USD)	MC,CC	Annual (mean)	35,143	25,129			
Bostrom (2007)^17^	USA (USD)	MC,ME,SC	Annual (mean)	45,800	22,200			
Mueller (2018)^31^	UK (GBP)	HPL	1y Pre + POD	3671	3110			
Espinosa (2020)^19^	USA (USD)	MC,ME	6m Pre + POD	41,104				
Frazer (2023)^23^	USA (USD)	MC,ME	Annual (mean)	22,738				
Hill (2005)^27^	USA (USD)	MC,ME	Annual (mean)		7839	14,387		
Fillit (2002)^21^	USA (USD)	MC,ME	Annual (mean)		13,274	31,177		
Ramos-Estebanez (2012)^37^	Spain (USD)	MC,CC	Annual (mean)			22,631		
Sicras (2005)^38^	Spain (EUR)	MC,DT,ME,SC	6m		8580	11,709		

*VD = Vascular Dementia; DLB = Dementia Lewy Body; PDD = Parkinson’s Disease Dementia; FTD = Frontotemporal Dementia.

*MC = Medical care; ME = Medication; CC = Community care; SS = Social care; HPL = Hospitalisation; TP = Transportation; DT = Diagnostic Test.

*m = month; y = year.

*All costs converted to USD using average 2025 exchange rates: 1 EUR = 1.06 USD, 1 GBP = 1.26 USD.

### Parkinson’s Disease Dementia (PDD)

Ten studies included PDD as part of a comparative analysis between dementia subtypes, with six reported findings specific to people with PDD. Unfortunately, none of these studies studied PDD as the primary group of interest, highlighting the gap in research on the HCU for PDD.

PDD face a higher burden of several health complications compared to other dementia types. Studies^13,22^ have found that PDD is associated with a greater frequency of cerebrovascular risk factors, stroke, falls and fractures, malignancies, hallucinations, and episodes of acute delirium. Forns et al. (2022) reported that PDD had the highest rates of malignancy (39.6%) and falls or fractures (38.5%) among all dementia subtypes, with a particularly high incidence rate for falls at 66.16 per 100 person-years (95% CI: 65.59-66.73). Moreover, PDD was associated with the highest rate of aspiration pneumonia (5.9%), while upper respiratory infections were more common in unspecified dementia (11.2%).^22^

Beyond physical health challenges, PDD also faces a significant decline in dental care utilisation and increased HCU. Fereshtehnejad (2018) reported that PDD had more severe dementia at baseline and showed a more rapid decline in dental care utilisation, particularly when the dementia was more severe or cognitive decline occurred faster during the follow-up period compared to AD.^20^ Multiple studies have reported that PDD exhibits significantly higher HCU compared to other dementia subtypes,^12^ including longer durations of hospital stay^13^ and an increased risk of hospital admission.^32^ Murman et al. (2002) further observed that PDD was more likely to be hospitalised than AD or Huntington’s disease (73% vs 57% and 47%, respectively) and also incurred higher direct healthcare costs.^33^ Key contributors to the elevated HCU in PDD include inpatient hospital care, outpatient or physician services, and long-term care, such as skilled nursing facilities.^15,32^

### Dementia With Lewy Bodies (DLB)

Seventeen studies included DLB, with eight reporting specific findings to the DLB cohort. Two have DLB as the primary group of interest,^19,39^ and six compared DLB and AD.^17,24,31,33,35,41^

DLB exhibit distinct patterns of HCU and clinical presentation when compared to other dementia subtypes. DLB exhibit a complex clinical profile, characterised by a higher burden of comorbidities^33,35^ and a greater likelihood of being diagnosed with multiple dementia types.^23^ They also experience a range of adverse clinical outcomes,^14,18,19,22,24^ including physical conditions such as falls, fractures, infections, pneumonia, dehydration, and motor symptoms, as well as neuropsychiatric symptoms including hallucinations, delusions, delirium, and sleep disorders, autonomic dysfunction and mood disturbances. Zhu et al. (2008)^41^ reported that DLB had significantly higher rates of psychotic symptoms (60%) and behavioural problems (64%) compared to those with AD, while Forns et al. (2022)^22^ noted that hallucinations were particularly prevalent in both DLB (38.0%) and PDD (35.5%). Desai et al. (2022)^15^ further found that DLB were more likely to be diagnosed with memory loss than those with PDD (47.9% vs 31.2%). Compared to AD, DLB also demonstrates greater impairments in functional domains, such as activities of daily living, occupational and recreational participation, and social relationships.^31^

Notably, fall-related injuries, including fractures and subdural haematomas, are more frequent in DLB and represent one of the leading causes of hospitalisation.^22,24^ Furthermore, neuropsychiatric symptoms, including hallucinations, delusions, and confusion, are more prevalent in DLB and contribute significantly to frequent hospital admissions.^30,39^ Spears et al. (2019)^39^ emphasised that neuropsychiatric symptoms—particularly hallucinations and confusion—were the most common causes of hospitalisation in DLB, accounting for 40% of admissions, followed by falls (24%) and infections (23%). Importantly, one-third of these admissions led to a transition to a higher level of care, and 15% resulted in death or hospice care.^39^ Despite exhibiting similar rates of cognitive decline to AD, DLB are more likely to be hospitalised and face higher mortality, particularly from fall-related injuries and bronchopneumonia.^24^ Numerous studies have demonstrated that DLB experience substantially higher psychosis-related HCU, which includes increased outpatient visits,^17,18^ more frequent general practitioner consultations due to dementia symptoms,^23^ greater use of informal services,^17,18^ and a higher rate of formal long-term care.^33^ Additionally, DLB often require more prescription medications,^17,23,33^ ambulance services,^18^ and emergency department visits.^15,18,22^ Several studies highlight the notably higher inpatient hospitalisation rates and prolonged lengths of stay in DLB.^18,23,31,35^ For instance, Frazer et al. (2023)^23^ found that hospitalisation costs and service usage for DLB were 61% higher than for other dementias. Murman et al. (2003)^33^ further reported that DLB experienced significantly more days of hospital care and required a greater number of prescription medications than other dementias. Oesterhus et al. (2020)^35^ observed that DLB had a markedly shorter time to their first hospitalisation (median 1.28 years; 95% CI: 0.93–1.67) compared to those with AD (median 2.32 years; 95% CI: 1.74–3.31) and spent more days in hospital (median 13 days vs 7 days). Within 5 years, 87% of DLB patients were expected to be hospitalised at least once, compared to 69% of AD patients. Furthermore, DLB had 84% more hospital days, an 86% higher hospitalisation hazard, and longer lengths of stay for unplanned admissions (7 vs 2 days), with a shorter median time to first hospitalisation (1.28 vs 2.32 years) than their AD counterparts. Interestingly, despite no significant differences in cognitive decline between AD and DLB, more DLB patients were hospitalised or died from fall-related injuries and bronchopneumonia compared to those with AD.^24^ Mueller et al. (2018)^31^ also reported that DLB experienced more frequent and prolonged hospitalisations, largely driven by poor physical health and neuropsychiatric symptoms.

### Vascular Dementia (VD)

Twenty studies included VD, the most researched non-AD subtype in this review. Seventeen analysed VD as a comparative cohort with other dementia subtypes, and three were comparative analyses between VD and AD. However, only 15 reported findings specific to VD.

Multiple studies have reported the highest prevalences of baseline comorbidities in VD,^22,28,29,42^ including both cerebrovascular and cardiovascular comorbidities,^21,27,38^ some uncorrelated with cardiovascular diseases such as anaemia and COPD,^12^ and frequencies of delusions.^22^ VD had a higher prevalence of clinical stroke history,^13,27,28,38^ hypertension,^38^ diabetes,^38^ and Parkinson’s disease^28^ compared to AD. Fillit and Hill (2002)^21^ reported that 70.0% of VD had cerebrovascular disease, compared to AD (34.6%) and controls (9.4%). Additionally, 42.9% of VD had congestive heart failure, substantially more than those with AD (23.9%) or controls (13.3%). Landi et al. (1998)^28^ confirmed a higher prevalence of a wide range of chronic medical conditions among VD, including stroke, coronary heart disease, diabetes, COPD, and arthritis. It is essential to note that dementia with cerebrovascular disease is preferentially diagnosed as VD, resulting in a higher prevalence of vascular risk factors in this group. Forns et al. (2022)^22^ and Chang et al. (2015)^13^ both found that VD patients had higher rates of acute events such as falls, hip fractures, and new strokes at baseline and were more likely to have three or more cerebrovascular-related risk factors compared with AD (OR 1.385). The odds of fall-related fracture were also significantly higher among VD patients compared with ODD (OR 4.302) and AD (OR 8.024).

VD consistently exhibited high levels of HCU compared to other dementia subtypes across a range of health service domains. Albrecht et al. (2018)^12^ reported that VD had the highest all-cause HCU rates, long-term care service usage, and the rates of disease-specific HCU three years pre-diagnosis compared to other dementia subtypes. Sicras et al. (2005)^38^ found that 91% of VD cohort attended some type of medical facility compared to 89% of those with AD; more supplemental examination was done for VD (2.0 vs 1.5) with more laboratory tests (57% vs 51%) requested, double the mean primary care follow up visits (14.7vs8.8), reference specialist visits (1.3 vs 1.1), and ED visits (0.6 vs 0.4) for VD compared to AD. Pisu et al. (2021) (36) highlighted the significant association between VD diagnosis and higher specialist visits. Chen et al. (2019) (15) highlighted similar findings, with VD having more annual ED visits (5.8 vs 4.2) and double ambulance usage (6.5 vs 3.8) compared to AD. High usage of HCU corresponded to the high proportion of the VD cohort living in long-term care and high levels of comorbidities at diagnosis.^12^ Landi et al. (1999)^28^ reported that, unlike AD, the level of cognitive impairment did not influence the risk of hospitalisation. VD had a high incident rate for nursing facility admissions^22^ and skilled nursing facility services compared to other dementia groups.^27^ Jaeger et al. (2021)^16^ reported that people who were assessed before the 1-year mark were more likely to be diagnosed with VD due to sudden and subacute onset, neuropsychiatric symptoms at presentation, rapid progression, and alcohol and antipsychotic use.

Higher HCU levels in VD were driven mainly by inpatient hospitalisation.^13,18,21,22,27-29,34,37,38,42^ Sicras et al. (2005)^38^ reported a higher proportion of hospitalisation in VD (27% vs 13%) with a higher average hospital stay (4.7 vs 2.4 days) compared to AD. Hill et al. (2005)^27^ reported similar findings with double the admission rate (0.83 vs 0.46), triple the length of stay per hospital admission (10.35 vs 3.59), and five more days per hospital admission (12.5 vs 7.8 days) compared to controls. Similarly, Fillit et al. (2002),^21^ Zilkens et al. (2009),^42^ Chang et al. (2015),^13^ and Chen et al. (2019)^18^ reported that annual hospitalisation rates and lengths of stay for VD were almost double compared to AD. Ramon-Estebanez et al. (2012)^37^ added that ischaemic events and hospital admissions due to non-neurological conditions were more common in VD, including falls,^22^ hip fractures,^13,34^ and pneumonia.^34^

### Patterns and Costs of Healthcare Utilisation

Fifteen studies included the cost and factors associated with higher HCU in people with non-AD (Table 5). Of these, only two focused on FTD, while four investigated PDD, six examined VD, ten explored DLB, and eleven addressed AD. All costs were converted to USD using average 2025 exchange rates: 1 EUR = 1.06 USD; 1 GBP = 1.27 USD.

Dauphinot et al. (2021)^14^ reported that PDD was the most expensive dementia subtype (EUR21,155;USD22,471), followed by DLB (EUR20,433;USD22,582) compared to AD (EUR10,444; USD11,073) in the first-year post-diagnosis. Henderson et al. (2022)^26^ similarly reported that PDD and DLB had the costliest weekly cost in year 1 (GBP650/USD825;GBP548/USD696), year 2 (GBP268/ USD340;GBP177/USD225), and year 3 (GBP513/USD65;GBP433; USD548). In comparison, FTD and VD had significantly increased costs overall at wave 1 (GBP69;GBP66), wave 2 (GBP123;GBP85), and wave 3 (GBP265;GBP131) (25). Chen et al. (2019)^18^ reported similar findings, with DLB having the highest annual cost per patient (USD22,514) followed by VD (USD21,002) and FTD (USD14,853) with a higher cost difference driven by annual hospitalisation rate and length of stay. In general, DLB had lower baseline HCU compared to PDD, including Emergency Department (ED) visits (DLB:51.0%;PDD:58.1%) and inpatient visits (DLB:31.1%;PDD:37.7%), totalling to USD15,903 (DLB) and USD21,040 (PDD) total medical costs annually.^15^ However, the lower healthcare cost of DLB compared to PDD was only significant during the first year post-diagnosis (USD27,496vsUSD31,080), and the mean total healthcare costs were similar between DLB and PDD three (USD23,124;USD23,681) and five years (USD22,977;USD22,583) post-diagnosis.^15^ Desai et al. (2022)^15^ reported that the main contributors to total medical costs for DLB and PDD were inpatient costs, followed by outpatient and skilled nursing facility costs. Henderson et al. (2019)^25^ confirmed significantly higher HCU and cost for PDD (GBP8,609;USD10,911) compared to AD (GBP3,484;USD4,415) driven by primary and community healthcare, social care, and medication. In contrast, DLB and VD had more ED visits (DLB:6.4;VD: 5.8) and ambulance use (DLB: 5.7;VD:6.5) annually compared to AD (ER:4.2; Ambulance:3.8) (15).

Six studies reported high HCU and costs in DLBs. Murman et al. (2003)^32^ reported that the DLB is significantly more expensive (USD25,311), with more days of formal long-term care and hospital care compared to an AD (USD11,557). Similarly, Zhu et al. (2008)^41^ estimated that the average annual costs of caring for DLB (USD35,143) and direct medical costs (USD12,081) were significantly higher than those for AD (USD25,129;USD8,027). Higher direct medical costs were accounted for, particularly by hospitalisation (USD6,937) and medications (USD3,185) in DLB compared to AD (USD3,096; USD2,900).^41^ Bostrom et al. (2007)^17^ also reported higher annual cost of care and HCU in DLB (USD45,800) compared to AD (USD22,200), with higher utilisation of outpatient care, informal care, community services and medication for DLB. Mueller et al. (2018)^31^ reported similar findings with DLB had significantly more hospital admissions, unplanned hospitalisations, and hospital days compared to AD during the year before and after dementia diagnosis. Mueller et al. (2018)^31^ also recorded that pre-diagnosis, DLB had a higher rate of infections, anaemia, and Parkinson’s disease as their primary causes of admission, resulting in a slightly higher hospitalisation cost (GBP2,896;USD3,671) compared to AD (GBP2,453;USD3,110). Additionally, the total average healthcare cost varied between insurance providers, with USD39,896 (commercial) and USD41,104 (Medicare) per person six months before and after DLB diagnosis.^23^ Total costs were driven primarily by inpatient costs that significantly increased following additional core features (e.g., fluctuating cognition, motor symptoms, and sleep behaviour disorder) identified.^19^ DLB with fluctuating cognition had the highest total healthcare costs, contributing to 47%–70% higher inpatient costs despite other core symptoms identified.^19^ Frazer et al. (2023)^23^ found that DLB were significantly more likely to utilise dementia-related services (79.2%;61.3%), including outpatient (72.0%;60.4%), ED (11.1%;7.7.%), and inpatient services (14.0%;9.1%) compared to those with other dementia subtypes with psychosis. In comparison between pre-and post-diagnosis, the average all-cause cost was similar, with DLB having significantly higher all-cause office visits (USD1,956;USD1,285), post-diagnosis all-cause office visits (USD1,497;USD1,065), and medication (USD6,616;USD4,515) totalling to USD22,738 compared to other dementia subtypes with psychosis (USD19,484).^23^

Four studies analysed patterns of HCU and cost in VD. VD had higher annual costs than AD (USD14,387; USD7,839), where higher costs were accounted for by the higher frequency of inpatient hospital and skilled nursing facility services.^27^ When compared to cerebrovascular disease without dementia, VD had lower costs for physician visits and prescription drugs but double the hospital admissions and triple the length of hospital stays.^27^ Fillit et al. (2002)^21^ reported that annual healthcare costs for VD were almost triple (USD31,177) compared to AD (USD13,274), driven by higher hospital costs (82%) and double the hospital days (13 days) for VD compared to AD (6 days). Ramos-Estebanez et al. (2012)^37^ reported similar findings regarding the increased average number of hospital admissions for VD (USD22,631), with ischemic events and non-neurological conditions being the most common cause of hospitalisation. Sicras et al. (2005)^38^ also reported higher HCU in VD compared to AD, especially for primary care, specialist, and ED visits. The per-patient cost was 36% higher in VD (EUR11,039; USD11,709) compared to AD (EUR8,086; USD8,580). Despite higher hospitalisation admission (27% vs 13%) and longer hospital stay (4.7 vs 2.4 days), VD had a lower cost per hospitalisation and higher mean cost per patient for medical visits, diagnostic tests, transportation, medical and caregiver costs compared to AD (38). Hill et al. (2005)^27^ also reported that VD had lower use of physical office visits and prescription drugs compared to AD.

## Discussion

While numerous studies have evaluated healthcare utilisation (HCU) in dementia^43–45^, this review is among the first to systematically synthesise evidence on patterns and predictors of HCU among individuals with non-Alzheimer’s dementia (non-AD), including dementia with Lewy bodies (DLB), frontotemporal dementia (FTD), vascular dementia (VD), and Parkinson’s disease dementia (PDD). Despite including 31 studies, the literature on HCU in non-AD populations remains limited, with evident disparities in subtype focus, country representation, and measurement methods. PDD and FTD were particularly underrepresented, and few studies prioritised VD or PDD as their primary focus, highlighting notable research gaps. Most studies originated from high-income countries, where better diagnostic and healthcare infrastructure may explain increased attention to these conditions. The majority were rated as “fair” due to inconsistent reporting and outcome measurement. HCU was often assessed through single indicators such as cost or isolated service use, limiting comparability. This underscores the need for standardised, composite measures of HCU that capture the complexity of service use across diverse healthcare systems, especially in low- and middle-income settings.

Findings across studies confirm the multifactorial nature of HCU in non-AD dementia. The diagnostic subtype alone was insufficient to explain service variation. Instead, increased utilisation and costs were commonly linked to greater comorbidity, frailty, and neuropsychiatric symptoms. Socioeconomic disadvantage, rurality, and minority ethnic status were consistently associated with higher HCU and extended hospital stays. Clinical factors such as cardiovascular, cerebrovascular, metabolic, and psychiatric comorbidities, reduced functional autonomy, delirium, polypharmacy, and caregiver burden also predicted higher service use. Dementia severity and behavioural symptoms were often stronger predictors than the diagnosis itself, reinforcing the importance of needs-based, person-centred approaches to care.

Specific non-AD subtypes demonstrated distinct patterns of HCU. FTD presented unique challenges due to early-onset behavioural symptoms that often led to frequent hospitalisations and longer lengths of stay. Despite these clinical complexities, FTD was associated with relatively low overall HCU and cost, possibly reflecting under-recognition of the condition or barriers to accessing appropriate behavioural and neuropsychiatric care. Lower Medicare coverage for FTD compared to other dementias such as VD may further exacerbate disparities. PDD was associated with a high healthcare burden due to physical comorbidities—such as stroke, falls, fractures, malignancies, and delirium—combined with rapid cognitive decline. This resulted in elevated use of inpatient and long-term care services, alongside reduced access to essential services such as dental care. DLB showed high reliance on unplanned hospitalisations, ambulance use, and treatment for falls and pneumonia, driven by both physical and neuropsychiatric symptoms. VD exhibited the highest all-cause HCU, primarily due to chronic cardiovascular and cerebrovascular conditions and high hospitalisation costs.

Altogether, this review offers a more nuanced understanding of HCU in non-AD dementia. Another significant but underexplored dimension to consider is the interplay between health and social care and the role of informal and paid caregivers in determining service use. Adequate social care provision, such as home support or community services, can help reduce reliance on acute or emergency healthcare by enabling individuals to remain safely in their own homes.^44^ Conversely, gaps in social care may result in increased hospital admissions or delayed treatment. Furthermore, care partners provide substantial support, yet their ability to do so is frequently limited by socioeconomic factors, employment demands, and access to care services.^46^ Notably, few included studies reported data on social care, limiting the ability to fully assess its impact on HCU in non-AD dementias. Addressing this gap should be a priority for future research to inform tailored, integrated, and multidisciplinary health and social care policies that effectively meet the medical and neuropsychiatric needs of these dementia subtypes while promoting equitable access and outcomes. Strengthening health systems with proactive, subtype-informed models could improve outcomes, optimise resource use, and ensure equitable, high-quality care for people with non-AD dementia.

## Limitation

Given the limited number of studies and countries represented, HCU patterns for people with non-AD may vary due to demographic, epidemiological, and healthcare system differences. Variations in healthcare organisation, funding models, access to services, and care pathways across countries and regions can significantly impact HCU patterns and outcomes. This heterogeneity complicates direct comparisons and generalisations across settings. Our focus on peer-reviewed English publications may have introduced publication bias and limited perspective diversity. Future research should consider grey literature and non-English sources to enhance generalisability. Although economic factors were considered, this review did not focus on cost-effectiveness; thus, databases like NHS EED and EconLit were not searched, possibly limiting economic insights. Dedicated reviews on economic evaluations in non-AD populations are needed to assess cost and affordability. Despite a comprehensive search strategy, some relevant studies may have been missed. Additionally, many included database studies inherently assume the accuracy of dementia diagnoses, which may affect the reliability of findings. More high-quality evidence is essential to clarify HCU patterns and associated factors.

## Conclusion

Despite the growing body of literature evidence demonstrating the unique needs of people with dementia, a significant yet smaller number of individuals with non-AD fail to receive optional support from the healthcare system to manage their conditions. Each non-AD subtype presents distinct and escalating healthcare challenges that demand proactive and targeted approaches that prioritise sustainable management of the condition and reduce unnecessary strains in the healthcare system. PDD’s intensive service needs and rapid cognitive decline underscore the urgency for early, integrated care models. DLB’s rising long-term costs highlight the need for sustained preventive measures to reduce costly emergency interventions. The behavioural complexities of FTD call for enhanced behavioural health integration, while VD’s high comorbidity burden points to a necessity for comprehensive, ongoing care strategies.
